# Pterygium: epidemiology prevention and treatment

**Published:** 2017

**Authors:** Sanjay Kumar Singh

**Affiliations:** Director, Eastern Regional Eye Care Programme, Biratnagar, Nepal.

**Figure:**
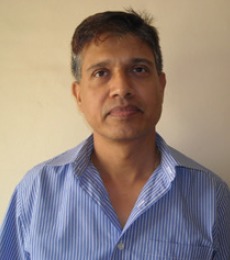
Prof Dr Sanjay Kumar Singh

## Epidemiology

Pterygium is a degenerative disorder of the conjunctiva. It is usually seen as a triangular fleshy fibrovascular proliferation from the bulbar conjunctiva onto the cornea, located mostly on the nasal side. Though it occurs worldwide, its prevalence is high in the “pterygium belt” between 30 degrees north and 30 degrees south of the equator.^1^ The prevalence of pterygium is reported to be 3% in Australians, 23% in blacks in United States, 15% in Tibetans in China, 18% in Mongolians in China, 30% in Japanese and 7% in Singaporean Chinese and Indians.^2–7^

In a population-based study from rural central India, prevalence of pterygium increased from 6.7±0.8% in the age group from 30–39 years to 25.3±2.1% in the age group of 70–79 years. Three population based studies have described the incidence of pterygium. Barbados eye study has described the nine year incidence of pterygium to be 11.6% (95% CI, 10.1–13.1), the Beijing Eye Study described the 10 year incidence of pterygium in the adult Chinese population to be 4.9%, and the five year cumulative incidence in Bai Chinese population in a rural community was 6.8% (95% CI, 5.2–8.4).^8–10^

## Risk factors and pathogenesis

These population-based studies suggest that cumulative ultraviolet light exposure due to outdoor occupation is a major risk factor for the development of pterygium. Other factors associated with pterygium development are age, being male and having dry eyes.^11–13^ Genetic factors, tumor suppressor gene p53 and other genes may be involved in the pathogenesis of pterygium.^14^

A study indicated a two-stage hypothesis for pterygium pathogenesis: initial disruption of the limbal barrier and progressive active “conjunctivalisation” of the cornea.^15^ Identification of Fuchs Flecks at the head of pinguecula, primary pterygium, recurrent pterygium, and macroscopically normal nasal and temporal limbus may represent precursor lesions to UV associated ocular surface pathology.^16^

## Prevention

Avoidance of environmental risk factors like sunlight, wind and dust by wearing UV rays protecting sunglasses and hat may prevent development of pterygium. These protective measures may help to prevent recurrence of pterygium after surgery. Similarly, wearing of eye safety equipment is recommended in environment exposed to chemical pollutants as a preventive measure for pterygium.

## Indication for surgery

The main indication for pterygium surgery is visual disturbance secondary to encroachment over the pupillary area or induced astigmatism. Other indications which can be considered are, restriction in eye movements, chronic redness and foreign body sensation, and cosmetic concerns.^17^

## Management

Surgery is the mainstay of treatment for pterygium causing visual disturbances. The primary complication of pterygium surgery is recurrence defined by regrowth of fibrovascular tissue across the limbus and onto the cornea. No uniformity of opinion exists regarding the ideal pterygium excision procedure associated with lowest recurrence rate. Bare sclera technique, which is widely used in the developing world for the ease and speed of surgery, is associated with high recurrence rates.^18^ Other adjunctive therapies combined with bare sclera technique have significantly reduced the recurrence rate (2% to 15%).^19^ Application of different agents like Strontium 90, Beta irradiation and cytotoxic drugs like Mitomycin-C and 5-Fluorouracil to the scleral bed have been tried but sight threatening complications like inflammatory scleritis, scleromalacia and loss of the eye have been occasionally reported.^20^ Amniotic membrane transplantation has been used after bare sclera technique with a reported recurrence rate of 4% to more than 60%.^21^,^22^ Currently, the most widely used procedure is pterygium excision with conjunctival autograft.^23^ Superior bulbar conjunctiva has been used widely since the early 1980s and is associated with recurrence rate of approximately 2% to 12% along with few complications.^24–26^ In the 1980s, Barraquer introduced the concept that removal of Tenon's layer may be important in reducing recurrence rate after pterygium removal as the tenon is the main source of fibroblasts.^27^ This was also emphasised by Solomon et al who combined this technique with Mitomycin-C application and amniotic membrane transplantation to achieve a low recurrence rate.^28^ A near zero recurrence rate with a good aesthetic result can be achieved by using Pterygium Extended Removal Followed by Extended Conjunctival Transplantation (P.E.R.F.E.C.T.).^29–31^ There is no ideal technique for conjunctival autografting which is safe, fast, easy and inexpensive. Various methods such as sutures, fibrin glue, autologous serum and electrocautery have been used for conjunctival autografting.^32^,^33^ Surgical steps for pterygium excision with conjunctival autograft that we have adopted at our hospitals under Eastern Regional Eye Care Programme in the eastern part of Nepal are as follows:

**Anaesthesia:** Peribulbar anaesthesia is preferable over the topical or subconjunctival to avoid pain during operation and to have smooth surgical procedure.

**Figure 1.**
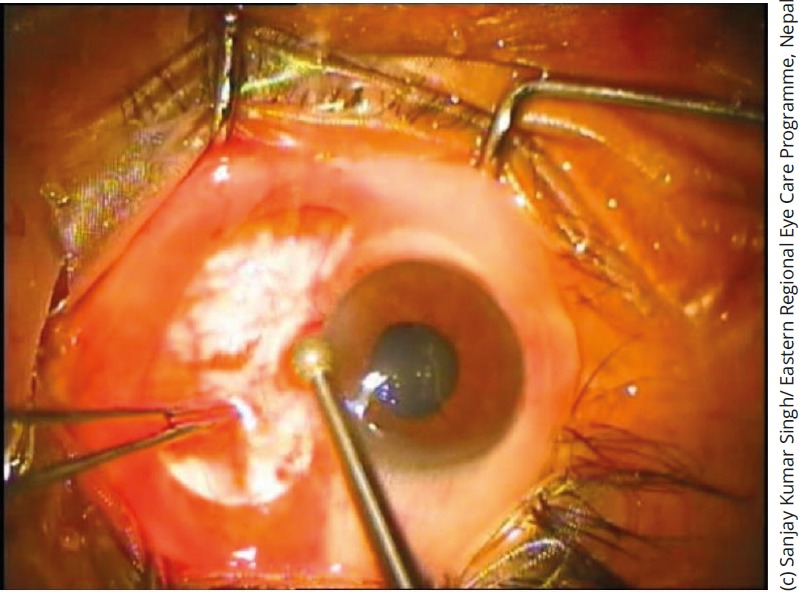
A diamond burr is used for smoothening of corneal surface

**Pterygium excision:** Pterygium body is excised carefully with conjunctival scissors and the head of pterygium can be removed from cornea by using a 15 degree Bard Parker blade. Tenons and subtenon tissue must be removed carefully as much as possible. Remaining pterygium tissues from over the corneal surface can be removed with a diamond burr.

**Conjunctival autograft preparation:** The conjunctival defect created by pterygium excision should be measured with a caliper and the superior bulbar conjunctiva should be marked by a marker. It is always preferable to use the marker to create exactly the same size of the graft. After marking, a subconjuctival injection of normal saline, around 2 ml, is injected on the superior bulbar conjunctiva to create the conjunctival balloon. A thin layer of conjunctival graft, devoid of tenons and subtenon tissue is prepared.

**Conjunctival grafting:** The thin conjunctival graft is placed with correct orientation on the area of the conjunctival defect created by pterygium excision. The marker helps to identify the correct orientation of the graft. The conjunctival graft can be sutured with the 8'0 Vicryl or 10'0 Nylon sutures or can be glued with fibrin glue.

**Figure 2.**
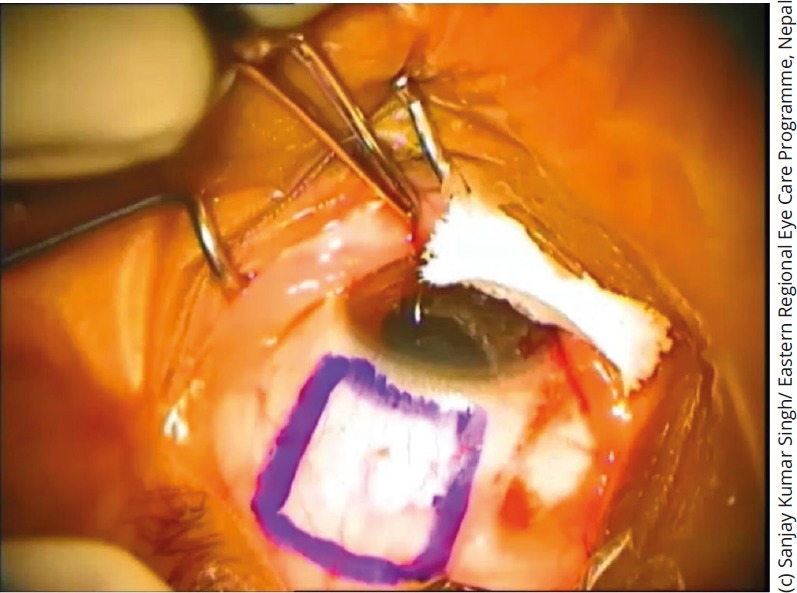
A conjunctival autograph marking

Conjunctival grafting with fibrin glue is a faster procedure and patients complain of less pain in the post-operative period.

**Post-operative management:** Antibiotic and steroid eye drops are given in tapering doses for one month.

## Conclusion

Many ophthalmologists think that pterygium is a trivial condition for which not much time should be expended in surgery and for which the financial remuneration is low.^34^ But the patients want a cure, free of recurrence with good cosmesis after surgery. Pterygium excision with conjunctival autograft with fibrin glue offers a low recurrence rate, good cosmetic outcome with a reasonable speed of the pterygium surgery.
